# Long noncoding RNA (lncRNA) metallothionein 1 J, pseudogene (MT1JP) is downregulated in triple-negative breast cancer and upregulates microRNA-138 (miR-138) to downregulate hypoxia-inducible factor-1α (HIF-1α)

**DOI:** 10.1080/21655979.2022.2077906

**Published:** 2022-06-15

**Authors:** Gangyue Wang, Yi Dong, Heng Liu, Nang Ji, Jilei Cao, Aihui Liu, Xin Tang, Yu Ren

**Affiliations:** Department of Breast, Beijing Obstetrics and Gynecology Hospital, Capital Medical University, Beijing, China

**Keywords:** Triple negative breast cancer, lncRNA MT1JP, miR-138, HIF-1α

## Abstract

Triple-negative breast cancer (TNBC) is a highly invasive subtype of breast cancer. This study explored the molecular mechanism and influences of metallothionein 1 J, pseudogene (MT1JP), microRNA-138 (miR-138), and hypoxia-inducible factor-1α (HIF-1α) on TNBC cell proliferation and migration. We confirmed TNBC cases by immunohistochemistry (IHC) staining. The expression of MT1JP in two types of tissue collected from 78 TNBC patients was detected by performing real-time quantitative fluorescence PCR (RT-qPCR). To further evaluate the relationship among MT1JP, miR-138 and HIF-1α, expression vectors of MT1JP and HIF-1α, as well as miR-138 mimic and inhibitor, were delivered into BT-549 cells. We observed that MT1JP was downregulated in TNBC. MT1JP was positively correlated with miR-138 but negatively correlated with HIF-1α in TNBC tissues. In TNBC cells, upregulation of miR-138 and downregulation of HIF-1α were observed after overexpression of MT1JP. In addition, overexpression of miR-138 resulted in downregulation of HIF-1α but did not affect the expression of MT1JP. Decreased proliferation rate of TNBC cells was observed after overexpression of MT1JP and miR-138. HIF-1α increased cell proliferation and migration. HIF-1α also suppressed the role of MT1JP and miR-138 in TNBC cell proliferation and migration. In conclusion, our findings demonstrated that MT1JP inhibited TNBC by regulating the miR-138/HIF-1α axis, indicating that MT1JP might serve as a biomarker or target for TNBC treatment.

## Graphical Abstract

**Figure:**
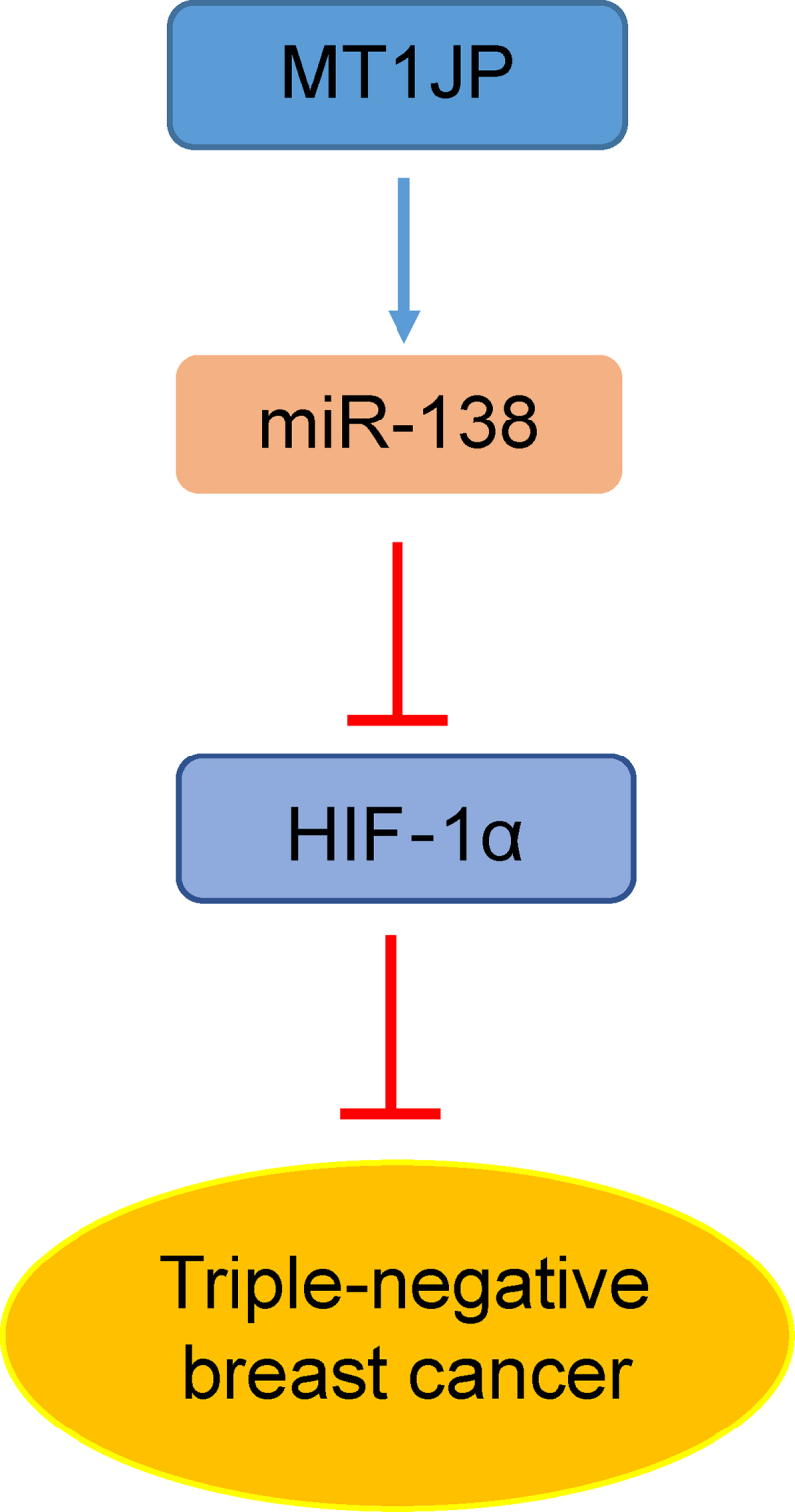

## Highlights

- MT1JP was positively correlated with miR-138
- miR-138 overexpression resulted in downregulated HIF‐ 1α
- T1JP and miR-138 overexpression decrease proliferation rate of TNBC cells
- HIF‐ 1α overexpression resulted in increased cancer cell proliferation

## Introduction

Breast cancer is the most common female malignancy [1]. Breast cancers are highly heterogeneous in nature and can be divided into at least five genetically distinct subtypes including Luminal A, Luminal B, HER2 overexpressing, and triple-negative breast cancer, which usually includes Basal-like and Claudin-low subtypes [2]. Triple-negative breast cancer (TNBC) is the most invasive and aggressive types among breast cancer subtypes [3]. Comparing to other subtypes of breast cancer, the survival of TNBC patients is even worse [4], due to the fact that TNBC does not respond to HER2 or estrogen receptor-directed treatments, and effective targeted therapies remain lacking [5,6]. The identification of potential biomarkers and therapeutic targets for TNBC requires better understanding of the mechanisms of triple negative breast cancer progression.

Studies on the occurrence and development of TNBC have identified a considerable number of dysregulated oncogenes or tumor suppressors involved in the pathogenesis of TNBC [7,8]. Long non-coding RNAs (lncRNAs, >200 nt) are also critical players in cancer biology [9,10]. LncRNAs encode no protein products but regulate gene expression at multiple levels [11]. Therefore, manipulating the expression of key lncRNAs may indirectly regulate the development of cancer [12]. However, the functions of most lncRNAs are unknown. LncRNA metallothionein 1 J, pseudogene (MT1JP) has been reported to be downregulated in cancers developed from liver, lung, colon and gastric cancer [13,14], suggesting its tumor suppressive role. MT1JP can modulate the p53 pathway by interacting with TIAR in liver, lung, colon and gagster cancer [13]. Zhu *et al*. demonstrated that MT1JP inhabited breast cancer cell proliferation, invasion, and cisplatin sensitivity by negatively regulating miR-24-3p [14]. MT1JP was also reported to inhibit biological activities of breast cancer cells *in vitro* and *in vivo* by regulating the miRNA-214/RUNX3 axis. MT1JP acts as a tumor suppressor by regulating miR-92-3p in breast cancer cells [15]. However, the function of MT1JP in TNBC is still unknown.

MicroRNAs are (miRNA) a class of conserved non-coding small RNA molecules with about 22 nucleotides in length and play vital roles in human cancers. MiR-138 has been reported to regulate metastasis and epithelial–mesenchymal transition (EMT) in breast cancer cells by targeting vimentin [16]. Moreover, it was reported that miR-138-5p inhibited the progression of colorectal cancer by regulating PD-L1 [17]. However, the role of miR-138 in TNBC development and progression remains unclear.

We hypothesized that MT1JP might regulate the biological functions of TNBC cells. This study aimed to explore the potential role of MT1JP in TNBC and the underlying molecular mechanisms. Our preliminary deep sequencing data showed that MT1JP was downregulated in TNBC and was correlated with miR-138 and hypoxia-inducible factor 1 (HIF-1α) (data not shown). It is known that miR-138 can target HIF-1α, which can transcriptionally activate more than 100 downstream genes in response to changes in oxygen, thereby promoting cancer progression [18]. The present study was carried out to investigate the role of MT1JP in TNBC.

## Materials and methods

### Research subjects

A total of 207 TNBC patients were admitted by Beijing Obstetrics and Gynecology Hospital, Capital Medical University between December 2015 and December 2018. This study enrolled 78 (age range from 27 to 64 years old, mean age 45.3 ± 5.2 years old) patients out of these 207 patients. Inclusion criteria: 1) new TNBC cases; 2) no family history of malignancies. Exclusion criteria: 1) recurrent TNBC; 2) TNBC combined with other clinical disorders; 3) any therapies (for any clinical disorders) were initiated before this study; 4) history of previous malignancies. Based on the staging methods established by AJCC, stage I–IV included 18, 29, 16 and 15 cases, respectively. The Ethics Committee of the aforementioned hospital approved this study. All patients were informed with the experimental procedures and the potential publication of this paper. All 78 TNBC patients signed the informed consent.

### Tissue biopsy

Breast biopsy was performed during the diagnosis of TNBC before any therapies were initiated. TNBC (cancer) and non-cancer tissues within 2 cm around tumor were collected from all the 78 patients (weight ranged from 0.09 to 0.17 g) through the dissection of biopsy by at least three experienced histopathologists. All tissues were subjected to histopathological examinations to make sure that correct tissues were collected.

### Cells and transient cell transfections

Human BT-549 and MDA-MB-231 cell lines were used in this study. BT-549 and MDA-MB-231 cells were purchased from the Cell Bank (Shanghai), China Academy of Sciences. RPMI-1640 medium was mixed with 10% fetal bovine serum (FBS) to cultivate BT-549 cells at 37°C with 5% CO_2_. MT1JP and HIF-1α expression vectors were constructed by Sangon (Shanghai, China) using pcDNA3.1 as the backbone vector. Negative control miRNA, miR-138 mimic, negative control inhibitor and miR-138 inhibitor were all purchased from Sigma-Aldrich (USA). BT-549 cells were collected when 70–90% confluence was achieved. MT1JP and HIF‐1α expression vectors (10 nM), empty pcDNA3.1 vectors (10 nM), negative control (NC) miRNA (35 nM), miR-138 mimic (35 nM), NC inhibitor (35 nM) and miR-138 inhibitor (35 nM) were transfected into 10^6^ cells using lipofectamine 2000 reagent (Invitrogen, USA). In this experiment, cells without transfections were used as the control cells (Control, C). Cells were collected at 24 h after transfections to perform the subsequent experiments.

### Real-time quantitative fluorescence PCR (RT-qPCR)

Total RNAs were extracted from tissues and BT-549 cells (collected at 24 h after transfections) using Ribozol (Sangon, Shanghai, China). Total RNAs were first digested by DNase I to remove genomic DNA and then used for cDNA preparation after the addition of poly (A) tail. qPCR was then performed to detect the expression of MT1JP, miR-138 and HIF-1α with 18S rRNA as the endogenous control [19]. qPCR reactions were repeated for 3 times, and Ct values were normalized to endogenous control using the 2^−ΔΔCT^ method. Expression levels of MT1JP were normalized to 18S rRNA, expression levels of HIF‐1α were normalized to GAPDH, and expression levels of miR-138 were normalized to U6. Primer sequences were as following: 5’-GAAATGGACCCCAACTACT-3’ (forward) and 5’-GTTCCCACATCAGGCACAGC-3’ (reverse) for MT1JP; 5’-GCCGCTGGAGACACAATCAT-3’ (forward) and 5’-GAAGTGGCTTTGGCGTTTCA-3’ (reverse) for HIF‐1α; 5’-CTTTGTGAAGCTCATTTCCTGGT-3’ (forward) and 5’-GTGGTTTGAGGGCTCTTACTC-3’ (reverse) for GAPDH; 5’-GTAACCCGTTGAACCCCATT-3’ (forward) and 5’-CCATCCAATCGGTAGTAGC-3’ (reverse) for 18S rRNA; 5’-GATAAAATTGGAACGATACAGAG-3’ (forward) and 5’-TCGATTTGTGCGTGTCATC-3’ (reverse) for U6; 5’-AGCUGGUGUUGUGAAUCAGG-3’ (forward) and poly (T) (reverse) for miR-138.

### Cell proliferation measurement

Cells were treated with 5% trypsin for 20 min before counting. Cells were cultivated at 37°C with 5% CO_2_, and CCK-8 (10 μl, Sigma-Aldrich) was added every 24 h. Finally, cell proliferation was analyzed by measuring OD values at 450 nM at 4 h after the addition of CCK-8. Control (C) group was set to value ‘1’, all other groups were normalized to C group [20].

### Transwell migration assay

Corning Transwell inserts were used for migration analysis. Cells were seeded into the non-serum medium in the upper chamber, while the lower chamber was added with complete medium to induce cell movement. Migrating cells were counted 24 h later after eosin staining. Control (C) group was set to value ‘1’, all other groups were normalized to C group [21].

### Western blot analysis

BT-549 cells (8 × 10^4^) were pretreated under the conditions of 1% O_2_/94% N_2_/5% CO_2_ for 12 h). Then, RIPA solution was used for protein isolation. Following denaturation, electrophoresis (10%, SDS-PAGE gel), gel transfer, blocking, and blotting were performed by incubation with GAPDH (ab37168, 1:1,200, Abcam) and HIF‐1α (ab26616, 1:1,200, Abcam) rabbit polyclonal primary antibodies at 4°C for 18 h or overnight. Following that, membranes were further incubated with IgG-HRP (MBS435036, 1:1,500, MyBioSource) goat anti-rabbit secondary antibody at 25°C for 2 h. ECL (Thermo Fisher Scientific) was then used to develop signals. Signals were then processed using Image J v1.46 software [22].

### Immunohistochemistry (IHC)

The ER, PR and HER-2 status of breast cancer specimens were detected by traditional pathological diagnosis. The molecular subtypes of these breast cancer patients were identified by immunohistochemical staining with ER, PR, and HER-2 according as previously reported [23].

### Nuclear/cytoplasmic fractionation

Nuclear/cytoplasmic fractionation was performed with PARIS Kit (Life Technologies, MA) following the manufacturer’s instructions. After the cell nuclear and cytoplasmic fractionating, the expression of MT1JP was detected by RT-qPCR and with GAPDH and U6 as the internal controls [24].

### Statistical analyses

All mean values were calculated using data from at least four biological replicates. Paired tissues (paired t test) and multiple groups (ANOVA) were compared. If any differences were observed, Tukey test was performed after ANOVA. Correlations were performed using linear regression. The 78 TNBC patients were divided into high and low MT1JP or miR-318 groups (n = 39, cutoff = median). Associations between patients’ clinical data and the expression levels of MT1JP or miR-318 were analyzed by Chi-squared test. Differences with *p* < 0.05 were statistically significant.

## Results

In this study, we aimed to explore the potential role of MT1JP in TNBC and the underlying molecular mechanism. We found that TNBC was significantly inhibited in TNBC tissues. Overexpression of MT1JP inhibited cell proliferation and migration. We further identified a significant positive correlation between MT1JP and the expression of miR-138, and a significant negative correlation between MT1JP and the expression of HIF-1α in tumor tissues. MT1JP and miR-138 could reduce the aggressiveness of TNBC cells by negatively affecting miR-138. In summary, our study confirmed the anti-tumor effect of MT1JP on TNBC, and MT1JP inhibited the progression of TNBC possibly by upregulating miR-138 and inhibiting HIF-1α. Our findings might provide potential therapeutic targets for TNBC treatment.

### MT1JP was downregulated with the development of TNBC

**Figure 1.**
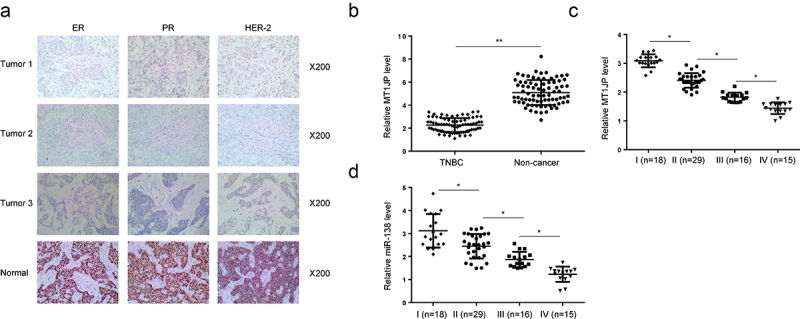
MT1JP was downregulated with the development of TNBC.

TNBC cases were identified by immunohistochemistry (IHC) staining of estrogen receptor (ER), progesterone receptor (PR) and epidermal growth factor receptor 2 (HER-2) (Figure 1a). Differential expression of MT1JP in TNBC was analyzed by RT-qPCR. The results showed that MT1JP was significantly downregulated in TNBC (Figure 1b, p < 0.05). Moreover, the expression levels of MT1JP (Figure 1c, p < 0.05) and miR-138-5p (Figure 1d, p < 0.05) were decreased from stage I to stage IV.

TNBC tissues were identified by ER, PR and HER-2 by using IHC (A). RT-qPCR analysis showed that MT1JP was downregulated in TNBC (B). MT1JP expression decreased from stage I to stage IV (C). MiR-138 expression in different clinical stage of TNBC patients (D). (*, *p* < 0.05).

### MT1JP was correlated with miR-138 and HIF-1α

**Figure 2.**
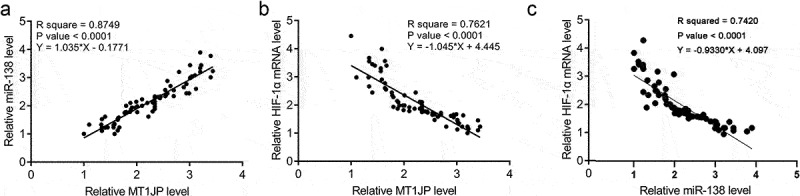
MT1JP was correlated with miR-138 and HIF-1α.

Our preliminary deep sequencing data showed that MT1JP was correlated with miR-138 and HIF-1α (Supplemental Fig. 1). In this study, the expression of miR-138 and HIF-1α in two types of tissue collected from 70 TNBC patients were also detected by performing RT-qPCR. Correlations among MT1JP, miR-138 and HIF-1α were analyzed. In TNBC tissues, MT1JP was positively and significantly correlated with miR-138 (Figure 2a). In contrast, MT1JP was negatively and significantly correlated with HIF-1α (Figure 2b). Moreover, miR-138 showed an inverse correlation with HIF-1α (Figure 2c). Chi-squared test analysis showed that MT1JP and miR-138 were closely associated with patients’ clinical stages and TNM classifications, but not age, family history and histological grade (Table 1).

Linear regression analysis showed that MT1JP was positively correlated with miR-138 (A), but negatively correlated with HIF-1α (B) in TNBC tissues.

**Table 1.** Chi-squared test analysis of the associations between patients’ clinical data and the expression levels of MT1JP or miR-138

Features	n	MT1JP		P	miR-138		P
		Low	High		Low	High	
Age(y)				>0.05			>0.05
< 45	40	19	21		18	22	
≥ 45	38	20	18		21	17	
Clinical stage				< .00001			< .00001
I–II	47	12	35		11	36	
III–IV	31	27	4		28	3	
T classification				< .00001			< .00001
T1-T2	53	17	36		16	27	
T3-T4	25	22	3		23	2	
N classification				0.000951			0.004619
N0-N1	50	18	32		19	31	
N2-N3	28	21	7		20	8	
M classification				0.006739			0.006739
M0	68	30	38		30	38	
M1	10	9	1		9	1	
Family history				> 0.05			> 0.05
No	70	34	36		34	36	
Yes	8	5	3		5	3	
Histological grade				> 0.05			> 0.05
G1	49	22	27		22	27	
G2-G3	29	17	12		17	12	

### MT1JP upregulated miR-138 to downregulate HIF-1α

**Figure 3.**
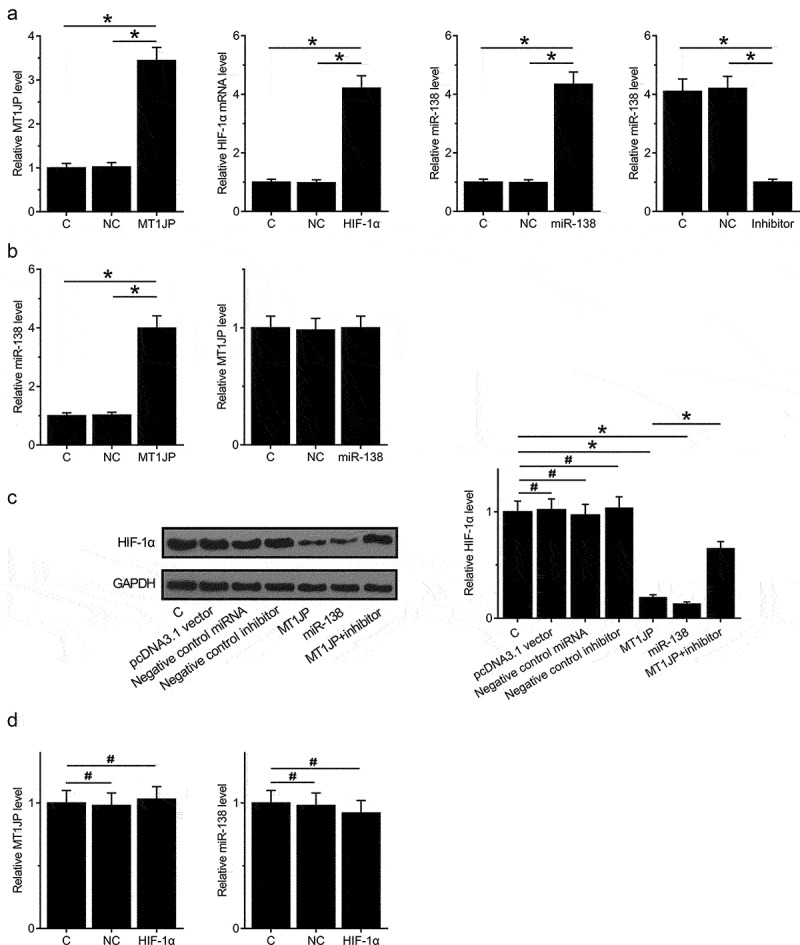
MT1JP upregulated miR-138 to downregulate HIF-1α.

Nuclear/cytoplasmic fractionation assay was performed to investigate the mechanism of how MT1JP regulates tumorigenesis. The results showed that MT1JP was primarily located in cytoplasm of TNBC cells (Supplemental Fig. 2A, *p* < 0.05). To further explore the relationship among MT1JP, miR-138 and HIF-1α, expression vectors of MT1JP and HIF-1α, as well as miR-138 mimic and inhibitor, were transfected into BT-549 cells. Comparing to C (control, cells with no transfection) and NC (negative control, empty vector or NC miRNA transfection), the expression levels of MT1JP, HIF‐1α and miR-138 were significantly altered at 24 h after transfection (Figure 3a, p < 0.05). In addition, upregulated expression of miR-138 was observed after overexpression of MT1JP, while no significant change in the expression levels of MT1JP was observed after overexpression of miR-138 (Figure 3b, p < 0.05). Moreover, downregulated expression of HIF-1α was observed after overexpression of miR-138 and MT1JP, and miR-138 inhibitor attenuated the effects of overexpression of MT1JP (Figure 3c, p < 0.05). In contrast, overexpression of HIF-1α did not affect the expression of miR-138 and MT1JP (Figure 3d). Moreover, RT-qPCR was used to detect the expression of HIF1a in TNBC transfected with MT1JP. It showed that overexpression of MT1JP inhibited the expression of HIF1a (Supplemental Fig. 2B, *p* < 0.05).

Comparing to C (control, cells with no transfection) and NC (negative control, empty vector or negative miRNA transfection), expression levels of MT1JP, HIF-1α and miR-138 were significantly altered at 24 h after transfection (A), suggestive of successful transfections. MT1JP overexpression resulted in the downregulation of miR-138, while no significant changes in expression levels of MT1JP was observed after miR-138 overexpression (B). In addition, downregulated HIF-1α was observed after miR-138 and MT1JP overexpression, and miR-138 inhibitor attenuated the effects of MT1JP overexpression (C). In contrast, HIF-1α, overexpression failed to affect miR-138 and MT1JP expression (D) (*, *p* < 0.05; #, *p* > 0.05).

### The MT1JP/miR-138/HIF-1α pathway regulated TNBC cell proliferation and migration

**Figure 4.**
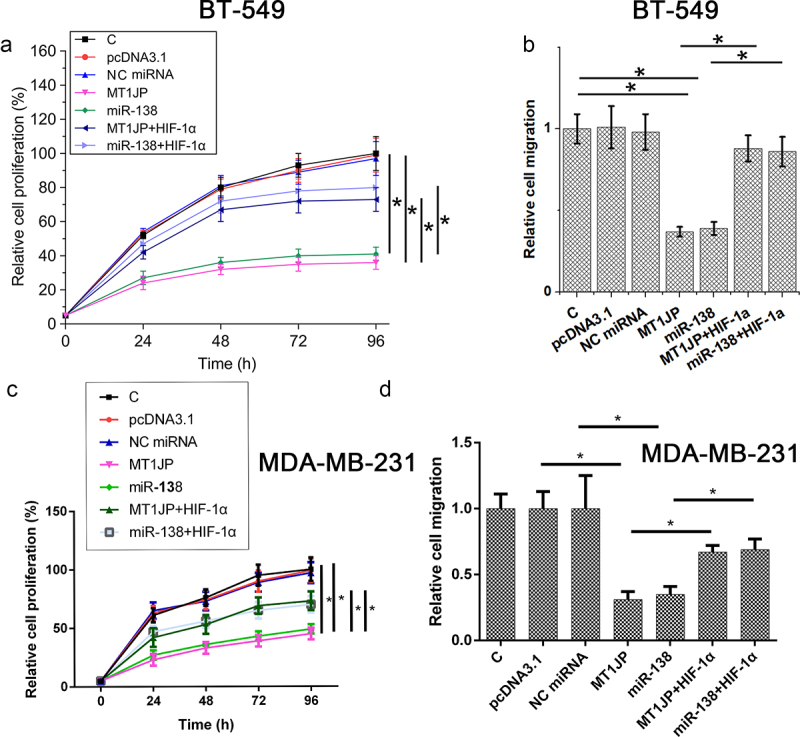
MT1JP/miR-138/HIF-1α pathway regulated TNBC cell proliferation and migration.

Cell proliferation assay and Transwell migration assay were performed at 24 h after transfections. Reduced TNBC proliferation and migration in BT-549 and MDA-MB-231 cells were observed after overexpression of MT1JP and miR-138. HIF‐1α increased cell proliferation and suppressed the role of MT1JP and miR-138 in TNBC cell proliferation and migration (Figure 4a–d, p < 0.05).

(A-D) Decreased proliferation and migration rate of BT-549 and MDA-MB-231 cells was observed after MT1JP and miR-138 overexpression. HIF‐1α overexpression resulted in increased cancer cell proliferation and migration. HIF‐1α also attenuated effects of MT1JP and miR-138 overexpression on TNBC cell proliferation and migration (*, *p* < 0.05).

## Discussion

A previous study has extensively investigated the expression of MT1JP in cancers developed from four types of organ [13]. Another recent study also showed that MT1JP was downregulated in breast cancer [14]. We analyzed the expression and function of MT1JP in TNBC and found that MT1JP was downregulated in TNBC and may play a tumor suppressive role by upregulating miR-138, which can direct target HIF-1α [25].

We showed the downregulation of MT1JP in TNBC cells, and the expression levels of MT1JP were decreased with the increase in clinical stages, indicating the involvement of MT1JP in TNBC. In a recent study, it was reported that overexpression of MT1JP suppressed the proliferation of gastric cancer cells by regulating the translation of p53 by interacting with TIAR [26]. Consistently, our study also observed suppressed proliferation of TNBC cells after overexpression of MT1JP. After synthesis, lncRNAs can be released into blood circulating system to achieve systemic trafficking. Therefore, detecting the expression levels of lncRNAs in blood derivatives may reflect cancer development [13,27].

The interactions between miRNAs and lncRNAs have been widely explored in previous studies [25,28]. It has been reported that lncRNAs can inhibit the roles of miRNAs by serving as their sponges [25]. However, the upregulation of miRNAs by lncRNAs has not been well studied. In the present study, we showed that MT1JP was an upstream positive regulator of miR-138. miR-138 plays a tumor suppressive role in cancer biology by targeting many oncogenes, such as HIF‐1α in ovarian cancer [17,18]. We observed downregulated expression of HIF‐1α in TNBC cells after overexpression of miR-138. Therefore, miR-138 may also target HIF‐1α in TNBC. Interestingly, we found that MT1JP downregulated HIF‐1α by upregulating miR-138 to inhibit TNBC cell proliferation and migration. Therefore, we characterized the MT1JP/miR-138/HIF‐1α axis as a novel axis involved in the regulation of TNBC cell proliferation. It is also worth noting that MT1JP can regulate p53 to exert its functions in cancer biology [26]. MiR-138 can also interact with the p53 signaling to play its biological roles [29]. Therefore, p53 or p53 pathway-related factors may mediate the interactions between miR-138 and MT1JP. Moreover, p53 can also directly regulate the degradation of HIF‐1α [30]. Therefore, it is also possible that MT1JP can also affect HIF‐1α accumulation through p53. Our future studies will try to test those possibilities. To the best of our knowledge, our study is the first to report a lncRNA that can upregulate miR-138. Previous studies mostly reported the role of lncRNAs as endogenous competing RNAs of miRNAs [31,32]. Our study reported a novel interaction between lncRNAs and miRNAs. This novel interaction may guide future studies on the function of lncRNAs and their interactions with miRNAs.

## Conclusion

MT1JP was downregulated in TNBC and overexpression of MT1JP may inhibit TNBC cell proliferation and migration by downregulating HIF-1α, which further upregulats miR-138. Our findings provide insights into potential therapeutic targets for TNBC treatment.

## Supplementary Material

Supplemental Material

## Data Availability

The analyzed data sets generated during the study are available from the corresponding author on reasonable request.

## References

[1] DeSantis C, Ma J, Bryan L, et al. Breast cancer statistics, 2013. CA Cancer J Clin. 2014;64(1):52–62. 24114568 10.3322/caac.21203

[2] Li P, Zhou B, Lv Y, et al. LncRNA HEIH regulates cell proliferation and apoptosis through miR-4458/SOCS1 axis in triple-negative breast cancer. Hum Cell. 2019;32(4):522–528. 31456112 10.1007/s13577-019-00273-1

[3] Boyle P. Triple-negative breast cancer: epidemiological considerations and recommendations. Ann Oncol. 2012;23(Suppl 6):vi7–12. 23012306 10.1093/annonc/mds187

[4] Sharma P, López-Tarruella S, García-Saenz JA, et al. Pathological response and survival in triple-negative breast cancer following neoadjuvant carboplatin plus docetaxel. Clin Cancer Res. 2018;24(23):5820–5829. 30061361 10.1158/1078-0432.CCR-18-0585 PMC6279513

[5] Denkert C, Liedtke C, Tutt A, et al. Molecular alterations in triple-negative breast cancer—the road to new treatment strategies. Lancet. 2017;389(10087):2430–2442. 27939063 10.1016/S0140-6736(16)32454-0

[6] Bianchini G, Balko JM, Mayer IA, et al. Triple-negative breast cancer: challenges and opportunities of a heterogeneous disease. Nat Rev Clin Oncol. 2016;13(11):674–690. 27184417 10.1038/nrclinonc.2016.66 PMC5461122

[7] Foulkes WD, Smith IE, Reis-Filho JS. Triple-negative breast cancer. New Engl J Med. 2010;363(20):1938–1948. 21067385 10.1056/NEJMra1001389

[8] Shah SP, Roth A, Goya R, et al. The clonal and mutational evolution spectrum of primary triple-negative breast cancers. Nature (London). 2012;486(7403):395–399. 22495314 10.1038/nature10933 PMC3863681

[9] Yang G, Lu X, Yuan L. LncRNA: a link between RNA and cancer. Biochimica Et Biophysica Acta -gene Regulatory Mechanisms. 2014;1839(11):1097–1109. 10.1016/j.bbagrm.2014.08.012 25159663

[10] Schmitt AM, Chang HY. Long noncoding RNAs in cancer pathways. Cancer Cell. 2016;29(4):452–463. 27070700 10.1016/j.ccell.2016.03.010 PMC4831138

[11] Engreitz JM, Haines JE, Perez EM, et al. Local regulation of gene expression by lncRNA promoters, transcription and splicing. Nature. 2016;539(7629):452. 27783602 10.1038/nature20149 PMC6853796

[12] Khorkova O, Hsiao J, Wahlestedt C. Basic biology and therapeutic implications of lncRNA. Adv Drug Del Rev. 2015;87:15–24. 10.1016/j.addr.2015.05.012 PMC4544752 26024979

[13] Liu L, Yue H, Liu Q, et al. LncRNA MT1JP functions as a tumor suppressor by interacting with TIAR to modulate the p53 pathway. Oncotarget. 2016;7(13):15787. 26909858 10.18632/oncotarget.7487 PMC4941277

[14] Zhu D, Zhang X, Lin Y, et al. MT1JP inhibits tumorigenesis and enhances cisplatin sensitivity of breast cancer cells through competitively binding to miR-24-3p. Am J Transl Res. 2019;11(1):245. 30787983 PMC6357327

[15] Wu H, Li S. Long non-coding RNA MT1JP exerts anti-cancer effects in breast cancer cells by regulating miR-92-3p. Gen Physiol Biophys. 2020;39(1):59–67. 32039825 10.4149/gpb_2019039

[16] Zhang J, Liu D, Feng Z, et al. MicroRNA-138 modulates metastasis and EMT in breast cancer cells by targeting vimentin. Biomed Pharmacother. 2016;77:135–141. 26796277 10.1016/j.biopha.2015.12.018

[17] Zhao L, Yu H, Yi S, et al. The tumor suppressor miR-138-5p targets PD-L1 in colorectal cancer. Oncotarget. 2016;7(29):45370. 27248318 10.18632/oncotarget.9659 PMC5216728

[18] Yeh YM, Chuang CM, Chao KC, et al. MicroRNA‐138 suppresses ovarian cancer cell invasion and metastasis by targeting SOX4 and HIF‐1α. Int J Cancer. 2013;133(4): 867–878 23389731 10.1002/ijc.28086

[19] He C, Liu Y, Li J, et al. LncRNA RPSAP52 promotes cell proliferation and inhibits cell apoptosis via modulating miR-665/STAT3 in gastric cancer. Bioengineered. 2022;13(4):8699–8711. 35322746 10.1080/21655979.2022.2054754 PMC9161851

[20] Ning M, Qin S, Tian J, et al. LncRNA AFAP-AS1 promotes anaplastic thyroid cancer progression by sponging miR-155-5p through ETS1/ERK pathway. Bioengineered. 2021;12(1):1543–1554. 33999777 10.1080/21655979.2021.1918537 PMC8806209

[21] Xu R, Zhang X, Xu Y, et al. Long noncoding RNA MST1P2 promotes cervical cancer progression by sponging with microRNA miR-133b. Bioengineered. 2021;12(1):1851–1860. 34034626 10.1080/21655979.2021.1921550 PMC8806230

[22] Meng C, Huang L, Fu X, et al. RAB27B inhibits proliferation and promotes apoptosis of leukemic cells via 3-Hydroxy butyrate dehydrogenase 2 (BDH2). Bioengineered. 2022;13(3):5103–5112. 35164665 10.1080/21655979.2022.2036903 PMC8973736

[23] Lv M, Xu P, Wu Y, et al. LncRNAs as new biomarkers to differentiate triple negative breast cancer from non-triple negative breast cancer. Oncotarget. 2016;7(11):13047–13059. 26910840 10.18632/oncotarget.7509 PMC4914340

[24] Ding J, Wang Q, Guo N, et al. CircRNA circ_0072995 promotes the progression of epithelial ovarian cancer by modulating miR-147a/CDK6 axis. Aging (Albany NY). 2020;12(17):17209–17223. 32877369 10.18632/aging.103668 PMC7521494

[25] Wu XS, Wang F, Li HF, et al. Lnc RNA‐PAGBC acts as a micro RNA sponge and promotes gallbladder tumorigenesis. EMBO Rep. 2017;18(10):1837–1853. 28887321 10.15252/embr.201744147 PMC5623869

[26] Xu Y, Zhang G, Zou C, et al. LncRNA MT1JP suppresses gastric cancer cell proliferation and migration through MT1JP/MiR-214-3p/RUNX3 axis. Cell Physiol Biochem. 2018;46(6):2445–2459. 29742512 10.1159/000489651

[27] Han L, Ma P, Liu S-M, et al. Circulating long noncoding RNA GAS5 as a potential biomarker in breast cancer for assessing the surgical effects. Tumor Biol. 2016;37(5):6847–6854. 10.1007/s13277-015-4568-7 26662314

[28] Paraskevopoulou MD, Hatzigeorgiou AG. Analyzing miRNA–lncRNA interactions. Long non-coding RNAs: Springer; 2016. p. 271–286. 10.1007/978-1-4939-3378-5_21 26721498

[29] Ye D, Wang G, Liu Y, et al. MiR‐138 promotes induced pluripotent stem cell generation through the regulation of the p53 signaling. Stem Cells. 2012;30(8):1645–1654. 22696098 10.1002/stem.1149

[30] Ravi R, Mookerjee B, Bhujwalla ZM, et al. Regulation of tumor angiogenesis by p53-induced degradation of hypoxia-inducible factor 1α. Genes Dev. 2000;14(1):34–44. 10640274 PMC316350

[31] Zhang ZK, Li J, Guan D, et al. A newly identified lncRNA MAR1 acts as a miR‐487b sponge to promote skeletal muscle differentiation and regeneration. J Cachexia Sarcopenia Muscle. 2018;9(3): 613–626 29512357 10.1002/jcsm.12281 PMC5989759

[32] Zhang H, Lu W. LncRNA SNHG12 regulates gastric cancer progression by acting as a molecular sponge of miR‑320. Mol Med Rep. 2018;17(2):2743–2749. 29207106 10.3892/mmr.2017.8143

