# Supplementary figures and images for: Long noncoding RNA (lncRNA) metallothionein 1 J, pseudogene (MT1JP) is downregulated in triple-negative breast cancer and upregulates microRNA-138 (miR-138) to downregulate hypoxia-inducible factor-1α (HIF-1α)
Source: Bioengineered. 2022 Jun 15;13(5):13718–27. doi: 10.1080/21655979.2022.2077906 (PMC9276039; doi:10.1080/21655979.2022.2077906)

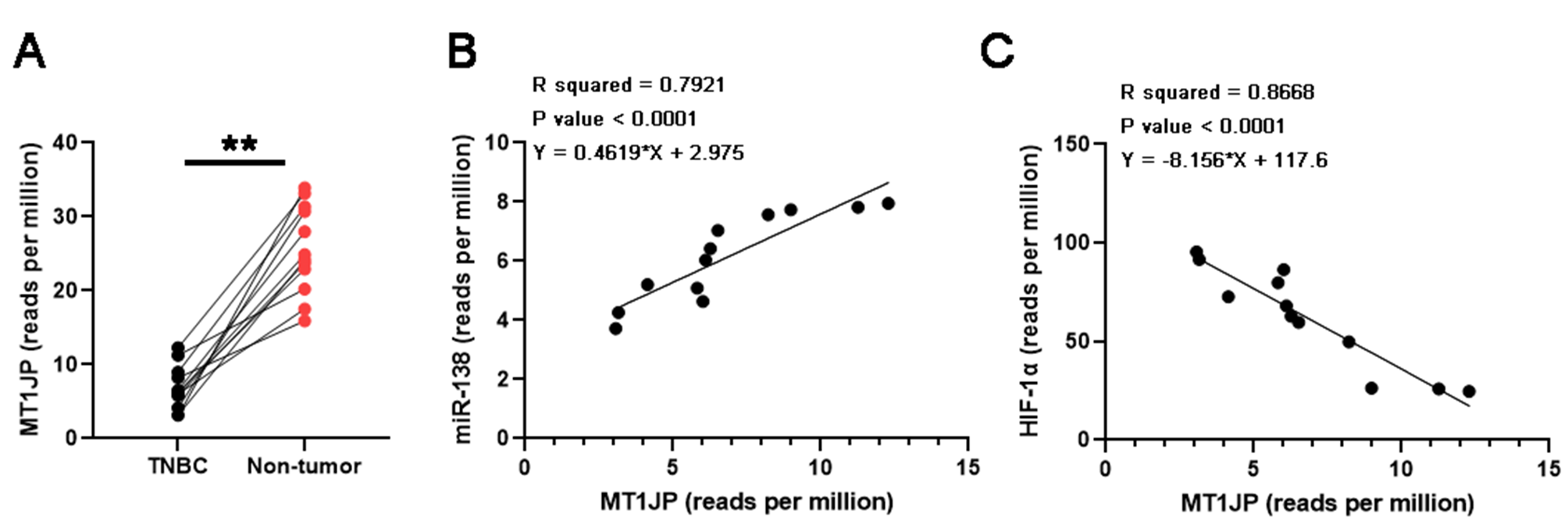

Supplement: Supplemental Material [file KBIE_A_2077906_SM3660.zip › Supplemental Figure 1.png]
